# Study of the Combined Effect of Maternal Tobacco Smoking and Polygenic Risk Scores on Birth Weight and Body Mass Index in Childhood

**DOI:** 10.3389/fgene.2022.867611

**Published:** 2022-05-12

**Authors:** Georgina Fuentes-Paez, Geòrgia Escaramís, Sofía Aguilar-Lacasaña, Sandra Andrusaityte, Anne Lise Brantsæter, Maribel Casas, Marie-Aline Charles, Leda Chatzi, Johanna Lepeule, Regina Grazuleviciene, Kristine B. Gützkow, Barbara Heude, Léa Maitre, Carlos Ruiz-Arenas, Jordi Sunyer, Jose Urquiza, Tiffany C. Yang, John Wright, Martine Vrijheid, Natàlia Vilor-Tejedor, Mariona Bustamante

**Affiliations:** ^1^ Endocrine Regulatory Genomics, Department of Experimental and Health Sciences, Universitat Pompeu Fabra (UPF), Barcelona, Spain; ^2^ CIBER Epidemiología y Salud Pública (CIBERESP), Madrid, Spain; ^3^ Departament de Biomedicina, Institut de Neurociències, Universitat de Barcelona (UB), Barcelona, Spain; ^4^ Childhood and Environment, ISGlobal, Barcelona, Spain; ^5^ Universitat Pompeu Fabra (UPF), Barcelona, Spain; ^6^ Department of Environmental Sciences, Vytautas Magnus University, Kaunas, Lithuania; ^7^ Climate and Environmental Health, Norwegian Institute of Public Health, Oslo, Norway; ^8^ Université de Paris Cité, Inserm, INRAE, Centre of Research in Epidemiology and StatisticS (CRESS), Paris, France; ^9^ Department of Preventive Medicine, Keck School of Medicine, University of Southern California, Los Angeles, CA, United States; ^10^ Inserm, CNRS, Team of Environmental Epidemiology Applied to Development and Respiratory Health, Institute for Advanced Biosciences, University Grenoble Alpes, Grenoble, France; ^11^ Genetics Unit, Universitat Pompeu Fabra (UPF), Barcelona, Spain; ^12^ CIBER Enfermedades Raras (CIBERER), Barcelona, Spain; ^13^ Fundació Institut Mar D'Investigacions Mèdiques (IMIM), Barcelona, Spain; ^14^ Bradford Institute for Health Research, Bradford Teaching Hospitals NHS Foundation Trust, Bradford, United Kingdom; ^15^ Barcelonaβeta Brain Research Center (BBRC), Pasqual Maragall Foundation, Barcelona, Spain; ^16^ Centre for Genomic Regulation (CRG), The Barcelona Institute for Science and Technology, Barcelona, Spain; ^17^ Department of Clinical Genetics, Erasmus Medical Center, Rotterdam, Netherlands

**Keywords:** gene by environment (GxE) interaction, maternal smoking, polygenic risk score, birth weight, body mass index, waist circumference, fat mass, children

## Abstract

**Background:** Maternal smoking during pregnancy has adverse health effects on the offspring, including lower birth weight and increased risk for obesity. These outcomes are also influenced by common genetic polymorphisms. We aimed to investigate the combined effect of maternal smoking during pregnancy and genetic predisposition on birth weight and body mass index (BMI)-related traits in 1,086 children of the Human Early Life Exposome (HELIX) project.

**Methods:** Maternal smoking during pregnancy was self-reported. Phenotypic traits were assessed at birth or at the age of 8 years. Ten polygenic risk scores (PRSs) per trait were calculated using the PRSice v2 program. For birth weight, we estimated two sets of PRSs based on two different base GWAS summary statistics: PRS-EGG, which includes HELIX children, and PRS-PanUK, which is completely independent. The best PRS per trait (highest *R* ^2^) was selected for downstream analyses, and it was treated in continuous or categorized into three groups. Multivariate linear regression models were applied to evaluate the association of the explanatory variables with the traits of interest. The combined effect was evaluated by including an interaction term in the regression models and then running models stratified by the PRS group.

**Results:** BMI-related traits were correlated among them but not with birth weight. A similar pattern was observed for their PRSs. On average, the PRSs explained ∼4% of the phenotypic variation, with higher PRS values related to higher trait values (*p*-value <5.55E-08). Sustained maternal smoking was associated with lower birth weight and higher BMI and related traits (*p*-value <2.99E-02). We identified a gene by environment (GxE) interaction for birth weight between sustained maternal smoking and the PRS-EGG in three groups (*p*-value interaction = 0.01), which was not replicated with the PRS-PanUK (*p*-value interaction = 0.341). Finally, we did not find any statistically significant GxE interaction for BMI-related traits (*p*-value interaction >0.237).

**Conclusion:** Sustained maternal smoking and the PRSs were independently associated with birth weight and childhood BMI-related traits. There was low evidence of GxE interactions.

## Introduction

Maternal tobacco smoking during pregnancy remains a great concern in public health, with 4.2–18.9% of European mothers smoking during the pregnancy period (Smedberg et al., 2014). Children of mothers who smoke during pregnancy have lower birth weights (BW) (Abraham et al., 2017) and an increased risk of developing obesity and metabolic problems in childhood (Behl et al., 2013; Rayfield and Plugge, 2017). A poor start in life, including low BW or being overweight in childhood, increases the risk of type 2 diabetes, cardiovascular disease, certain cancers, and mental health problems in later life (Weihrauch-Blüher and Wiegand, 2018). This is known as the Developmental Origins of Health and Disease (DOHaD) theory which postulates that adverse intrauterine environments promote adaptations in the developing fetus that lead to health problems in adulthood (Suzuki, 2017). BW and obesity are considered complex traits as they are influenced by genetic polymorphisms, environmental factors, and their interactions (GxE) (Yengo et al., 2018; Warrington et al., 2019; Sulc et al., 2020). Genome-wide association studies (GWAS) have shown that complex traits are highly polygenic: they are influenced by thousands of single nucleotide polymorphisms (SNPs) with slight effects that globally explain a substantial proportion of the phenotypic variation (Visscher et al., 2017). For instance, more than 211 genome-wide significant independent SNPs have been identified for own BW (Warrington et al., 2019) and 941 for body mass index (BMI), in both cases explaining ∼6% of the phenotypic variation (Yengo et al., 2018). SNP heritabilities (i.e., the proportion of phenotypic variance explained by all measured or imputed SNPs) for these two traits have been estimated at 28.5 and 22.4%, respectively. The polygenicity of complex traits has stimulated the development of disease risk prediction biomarkers based on the aggregation of several SNPs. Polygenic risk scores (PRSs) are a way to summarize genetic predisposition for a given trait (Choi et al., 2020). PRSs are usually computed as a weighted sum score of the number of risk alleles, using effect sizes from reference genome-wide association studies as the weights. Therefore, the computation of PRSs requires two input data sets: the base or reference data, which consists of the summary statistics of genetic variants from the published GWAS of the trait of interest, and the target data, which consists of genetic information from the individuals of the study population. The individuals in the reference and the target datasets must be non-overlapping and of the same genetic ancestry (Mostafavi et al., 2020). PRSs are applied to an individual’s disease risk prediction with the final goal of providing personalized preventive strategies and treatments (Lewis and Vassos, 2020; Wray et al., 2021). Previous studies have shown that PRSs for BW and BMI, calculated using different statistical approaches and including different numbers of SNPs, explain between 2 and 11% of the phenotypic variation (Horikoshi et al., 2016; Khera et al., 2019; Xie et al., 2020; Hüls et al., 2021; Odintsova et al., 2021). Despite this, PRSs are not deterministic. For example, it was found that 17% of the subjects in the top decile of a PRS that included 2.1 million SNPs were not overweight or obese (Khera et al., 2019). Thus, additional factors should be considered for accurate prediction.

Another application of PRSs is the investigation of pleiotropy. Pleiotropy is defined as the shared influence of a genetic variant on more than one unrelated phenotype and is common in complex traits (Visscher et al., 2017). For instance, BW SNPs are also associated with height, glucose metabolism, or blood pressure, which can explain, in part, the link between low BW and later cardio-metabolic problems. In particular in the case of BW, but also for other traits where the phenotype is influenced by the direct effect of the fetal genotype and the indirect effect of the maternal genotype that controls the intrauterine environment, pleiotropy has an additional degree of complexity (Warrington et al., 2019). Through a phenotype-wide association study (PheEWAS), a PRS for BMI was found to be related to 40 disease outcomes, spanning endocrine/metabolic, circulatory, and other disease groups (Dashti et al., 2022).

Finally, PRS can also be used to stratify the environmental risk to disease by conducting gene by environmental (GxE) interaction analyses (Domingue et al., 2020; Lewis and Vassos, 2020). Some studies have shown the interaction of PRSs for BMI with specific environmental factors such as parental education (Hüls et al., 2021), with obesity lifestyle index (Dashti et al., 2022), or with all potential environmental exposures estimated from the data (Sulc et al., 2020).

In this study, we aimed to investigate the combined effect of maternal smoking during pregnancy and genetic predisposition on BW and BMI-related traits. We calculated several PRSs for these traits in children of the Human Early Life Exposome (HELIX) project and then tested their GxE interactions with maternal smoking during pregnancy.

## Materials and Methods

### Study Population

HELIX (https://www.projecthelix.eu/) is a collaborative project across six established and ongoing longitudinal population-based birth cohort studies in six European countries: EDEN - Étude des Déterminants pré et postnatals du développement et de la santé de l’Enfant (France); Rhea - The Rhea Mother–Child Study in Crete (Greece); KANC - Kaunas Cohort (Lithuania), MoBa - Norwegian Mother, Father and Child Cohort Study (Norway), INMA - INfancia y Medio Ambiente (Spain), and BiB - Born in Bradford (United Kingdom) (Maitre et al., 2018). In summary, HELIX aims to investigate the effects of the early-life exposome on child health, identify determinants and exposure patterns, understand molecular mechanisms, and assess the role of genetic background. All the participants in the study signed an ethical consent, and the study was approved by the ethical committees of each study area.

PRS were calculated in the 1,155 European ancestry children from the HELIX project with genetic data, and the GxE interaction analyses were performed in 1,086 children with genetic, phenotypic, and exposure data **(** Supplementary Figure S1 **).**

### Maternal Tobacco Smoking in Pregnancy and Second-Hand Smoke in Childhood

Maternal tobacco smoking was self-reported by the mother during different trimesters of pregnancy, from which we created two variables: any and sustained maternal smoking. Any maternal smoking was categorized as “smokers” (mothers who smoked at any point during pregnancy) and “non-smokers” (mothers who did not smoke at all during pregnancy). Sustained maternal smoking was categorized as “non-smokers,” “non-sustained smokers” (mothers who smoked only during the first trimester), and “sustained smokers” (mothers who smoked until the third trimester).

Exposure to second-hand smoke in childhood was assessed through a harmonized questionnaire administered to the parents at the age of ∼8 years. Children were classified as “exposed” if they were exposed to second-hand smoke at home or in other indoor spaces and as “unexposed” if they were not exposed at all.

### Phenotypic Traits

BW (in grams) was collected as part of the study protocol of each cohort and harmonized in the context of the European Study of Cohort for Air Pollution Effects (ESCAPE) project (Agier et al., 2021). Whenever possible, gestation duration (in weeks) was defined as the interval between the start of the last menstruation and delivery; when the date of the last menstruation was missing, ultrasound-based estimates were used; when both measures were missing, obstetrician estimates were used. BW was converted in gestational age- and sex-standardized z-scores using the INTERGROWTH-21st reference curves (Villar et al., 2014). Six individuals had gestational age >300 days, which is outside the reference curves, and thus, for them, we could not calculate zBW.

At the age of ∼8 years, following a harmonized protocol across cohorts, anthropometry was assessed (Vrijheid et al., 2020). Children were asked to be in light clothing and without shoes, and then, height to the nearest 0.1 cm was measured with a stadiometer, and weight (in kg) was measured with a digital weight scale. BMI (in kg/m^2^) was calculated and converted into age- and sex-standardized z-scores (zBMI) using the international World Health Organization (WHO) reference curves (De Onis and Lobstein, 2010). Waist circumference (WC, in cm) was measured in a standing position, at the high point of the iliac crest at the end of a gentle expiration, using a metric tape and recorded in duplicate (Seca 201, Seca Corporation). Bioelectric impedance readings were performed with the Bodystat 1500 (Bodystat, Douglas, Isle of Man) equipment after 5 min of lying down. The proportion of fat mass (FM) was calculated using published age- and race-specific equations validated for use in children (Clasey et al., 2011). Using the distribution of the full study population combining all HELIX cohorts, we calculated age- and sex-standardized z-scores for waist circumference (zWC) and proportion of fat mass (zFM).

### Genome-Wide Genotyping, Quality Control, and Imputation

Child peripheral blood DNA samples were collected at the age of ∼8 years. Genome-wide genotyping was performed using the Infinium Global Screening Array (GSA) MD version 1 (Illumina) at the Human Genomics Facility, Erasmus MC (*HuGe-F*). Genotype calling was performed using the GenTrain2.0 algorithm based on a custom cluster file implemented in the GenomeStudio software and annotation with the GSAMD-24v1-0_20,011,747_A4 manifest.

Sample quality control was performed using the PLINK program (Purcell et al., 2007). The following filtering was applied for sample quality control: sample call rate <97% (n = 43), sex inconsistencies (n = 8), heterozygosity (>3 standard deviations) (n = 0), relatedness (sharing more than 18.5% of alleles) (n = 10), and duplicated samples (n = 19). The Peddy program was used to predict the ancestry from GWAS data and contrasted with self-reported ethnicity (Pedersen and Quinlan, 2017). Discordant samples were filtered out (n = 12). The following filtering was applied for the variant quality control: variant call rate <95% (n = 4,046), variants in non-canonical pseudoautosomal region (PAR) (n = 47), variants with minor allele frequency (MAF) < 1% (n = 178,017), and variants not in Hardy–Weinberg equilibrium (HWE), thus with a *p*-value <1E-06 (n = 913).

Genome-wide genotype imputation was performed with the Imputation Michigan server using the Haplotype Reference Consortium (HRC) cosmopolitan panel, version r1.1 2016. Before imputation, PLINK GWAS data were converted into *VCF* format, and variants were aligned with the reference genome. The phasing of the haplotypes was performed with Eagle v2.4 and the imputation with minimac4. Chromosome X was imputed, including PAR and non-PAR regions. In total, we retrieved 40,405,505 variants after imputation. Several quality control filters were applied to the imputed dataset: 1) imputation accuracy (*R* ^2^) <0.9, 2) MAF <1% and 3) HWE *p*-value <1E-06, giving rise to a final post-imputation dataset consisting of 1,304 samples and 6,143,757 variants (human genome build GRCh37/hg19 and plus strand). The first 20 principal components were computed from the GWAS data of European ancestry children using PLINK with the LD clumping option.

### Polygenic Risk Score Calculation and Validation

PRSs for BW, BMI, WC, and FM were computed for 1,155 children of the European ancestry using the PRSice v2 program and the imputed and quality controlled genetic data (Choi and O’Reilly, 2019). Summarized results of reference GWAS (base data) were retrieved from the Early Growth Genetics (EGG) consortium and from the PanUK Biobank—European population (Supplementary Table S1)**.** PRSs were calculated operating with the average score method, which computes the score as a sum of the summary statistics for the effective allele multiplied by the number of effective alleles observed, divided by the number of alleles included in the PRS. SNP clumping was set at *r* ^2^ > 0.1, and the rest of the arguments were left as default in PRSice v2. For each trait, we calculated 10 PRSs based on ten different *p*-value thresholds (Pt) of the base GWAS for SNP inclusion: Pt = {0.00000005, 0.000005, 0.0001, 0.001, 0.01, 0.05, 0.1, 0.2, 0.5, and 1}. For BW, we computed two sets of 10 PRSs: one using the EGG base GWAS (which includes around 25% of the HELIX children) (PRS-EGG) and the other using the PanUK Biobank base GWAS (with no overlap of individuals) (PRS-PanUK).

The optimal PRS for each trait among the 10 PRSs calculated with different *p*-value thresholds (Pt) was selected using PRSice v2. In particular, linear regression models were computed between the phenotypic trait and each PRS adjusting for the 10 first GWAS principal components (PCs). The PRS with the highest model-fit (*R* ^2^) was considered the best and used in downstream analyses. To control for overfitting due to parameter optimization (i.e., 10 Pt tested), the association between the best PRS and the phenotypic trait was corrected by performing 10,000 permutations of the phenotype and calculating an empirical *p*-value.

Finally, the best PRSs were centered and scaled by subtracting the mean and divided by the SD. They were analyzed as continuous variables and categorized into three groups: PRS-low (<25% percentile), PRS-mid (from 25 to 75% percentile), and PRS-high (>75% percentile). PRS was constructed based on the weights of the base GWAS; thus, the PRS group representing the highest genetic risk for zBW is the PRS-low, while for the other traits is the PRS-high group.

### Association of Maternal Smoking With Phenotypic Traits and Its Interaction With the PRSs

Frequencies (for categorical variables) and means and SDs (for continuous variables) were calculated. The cross-correlation of the PRSs and phenotypic traits was calculated with Pearson’s correlation coefficients and represented in heatmaps with the corrplot R package (Wei et al., 2017).

To test the association of the PRS and maternal smoking during pregnancy with phenotypic traits in the selected 1,086 children of the study, we fitted several linear regression models adjusted for covariates:

Phenotypic trait = PRS-continuous + any maternal smoking during pregnancy + covariates
Phenotypic trait = PRS-continuous + sustained maternal smoking during pregnancy + covariates
Phenotypic trait = PRS-groups + any maternal smoking during pregnancy + covariates
Phenotypic trait = PRS-groups + sustained maternal smoking during pregnancy + covariates

Covariates for zBW were sex, gestational age, 10 first GWAS PCs, and maternal education. Covariates for zBMI-related traits were 10 first GWAS PCs, maternal education (in three levels), and second-hand smoke in childhood. Maternal education, which is associated with socioeconomic status and maternal age, was included as a covariate as it is a potential confounder for maternal smoking during pregnancy.

Effect sizes are expressed as the change in the z-score of the phenotypic trait (where each unit represents one standard deviation from the mean considering age and sex), by maternal smoking status (non-smokers as reference), by PRS group (PRS-low as reference), or by one standard deviation of the PRS (when treated in continuous). The effect modification of the PRSs on the association between maternal smoking and the phenotypes was tested by including an interaction term between these variables in the linear regression model:

Phenotypic trait = PRS-continuous + any maternal smoking during pregnancy + PRS-continuous * any maternal smoking during pregnancy + covariates
Phenotypic trait = PRS-continuous + sustained maternal smoking during pregnancy + PRS-continuous * sustained maternal smoking during pregnancy + covariates
Phenotypic trait = PRS-groups + any maternal smoking during pregnancy + PRS-groups * any maternal smoking during pregnancy + covariates
Phenotypic trait = PRS-groups + sustained maternal smoking during pregnancy + PRS-groups * sustained maternal smoking during pregnancy + covariates

Partial F-tests were used to determine statistically significant differences between models with and without the interaction term (global interaction *p*-value). For statistically significant GxE interactions, models stratified by the PRS group were run.

Finally, we performed a series of sensitivity analyses to explore the effect on zBW of excluding cohorts included in the EGG-based GWAS summary statistics (INMA and MoBa) and of excluding non-preterm children. We also tested the effect on zBMI-related traits by not adjusting the models for childhood second-hand smoke and by excluding children unexposed to second-hand smoke.

All the analyses were conducted in R (version 4.0.3), and scripts can be found in a GitHub repository (https://github.com/georginafp/analysis_GxE).

## Results

### Description of the Study Population

The description of the main variables of the HELIX children is shown in Table 1. All the children were of European ancestry and were distributed across six ongoing European birth cohorts. In the study sample, 45.8% were females, the mean gestational age was 39.6 ± 1.6 weeks (4.9% preterm children), the mean age at the HELIX visit was 8.1 ± 1.5 years, and 52.5% were born to mothers with high educational attainment. Mean (standard deviation—SD) of BW and BMI were 3,392.9 (490.8) grams and 17.03 (2.63) kg/m^2^, respectively. For the analyses, phenotypic traits were sex- and age-standardized using international reference curves (zBW and zBMI) or own distributions (zWC and zFM). In total, 16.9% of the mothers reported having smoked at some point during pregnancy, and 9.7% were classified as sustained smokers as they smoked during the whole pregnancy period. Childhood exposure to second-hand smoke at home or other places was 36.8%. The correlation between pregnancy and childhood exposure was ∼0.4 and has been described elsewhere in detail (Vives-Usano et al., 2020). The description of the main variables by cohort is shown in Supplementary Table S2.

**TABLE 1:** Descriptive of the HELIX children (N = 1,086).

Variable	N (%) or mean (SD)
Cohort	
BIB (United Kingdom)	66 (6.1%)
EDEN (France)	135 (12.4%)
KANC (Lithuania)	193 (17.8%)
MoBa (Norway)	236 (21.7%)
RHEA (Greece)	185 (17.0%)
INMA (Spain)	271 (25.0%)
Sex	
Males	589 (54.2%)
Females	497 (45.8%)
Gestational age (weeks)	39.6 (1.6)
Preterm birth (<37 complete weeks)	52 (4.9%)
Birth weight (g) (BW)	3392.9 (490.8)
Birth weight (z-score) (zBW)	0.310 (0.985)
Age at assessment (years)	8.1 (1.5)
Body mass index (kg/m^2^) (BMI)	17.03 (2.63)
Body mass index (z-score) (zBMI)	0.460 (1.188)
Waist circumference (cm) (WC)	59.20 (7.72)
Waist circumference (z-score) (zWC)	0.007 (0.941)
Fat mass (g) (FM)	5.36 (2.81)
Fat mass (z-score) (zFM)	−0.062 (0.940)
Any maternal smoking in pregnancy	
Non-smokers	903 (83.1%)
Smokers (non-sustained and sustained)	183 (16.9%)
Sustained maternal smoking in pregnancy	
Non-smokers	903 (84.5%)
Non-sustained smokers	63 (5.9%)
Sustained smokers	104 (9.7%)
Second-hand smoke in childhood	
Unexposed	672 (63.2%)
Exposed	391 (36.8%)
Maternal education level	
High	570 (52.5%)
Middle	383 (35.3%)
Low	133 (12.2%)

N, sample size; SD, standard deviation. zBW and zBMI were calculated using international (gestational) age- and sex-adjusted reference curves; zFM and zWC were calculated subtracting the population mean and dividing by the SD, considering age and sex. Sample size is 1,086 for all variables, except for sustained maternal smoking in pregnancy (N = 1,070; 16 mothers had missing information on tobacco smoking in some trimesters), second-hand smoke in childhood (N = 1,063), preterm (N = 1,063), BW and zBW (N = 1,080), BMI and zBMI (N = 1,063), WC and zWC (N = 1,060), and FM and zFM (N = 1,052).

### Description of the PRSs and Their Association With the Phenotypic Traits

**FIGURE 1:**
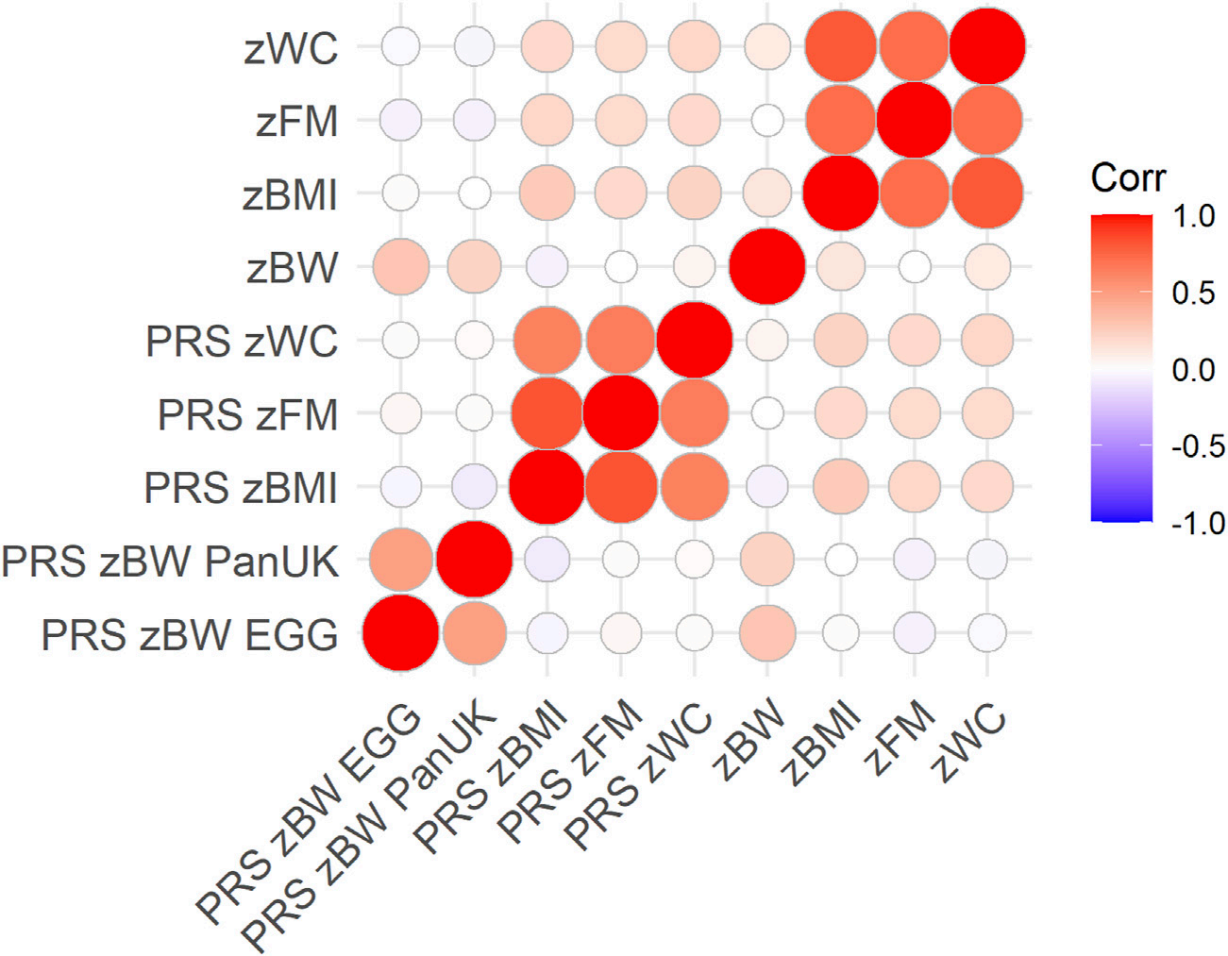
Pearson’s correlation coefficients across PRSs and phenotypic traits, considering only complete pairwise observations (N = 1,066). The color intensity and size of the circles indicate the degree of correlation.

Ten PRSs were calculated per phenotypic trait for the 1,155 HELIX children. The distribution of the PRSs, the total phenotypic variation explained (*R* ^2^), and the associations of the best PRS in quantiles are shown in Supplementary Figure S2. The best PRS for each trait was selected for downstream analyses. They contained 3,316, 18,563, 60,993, 9,203, and 62,011 SNPs and explained 4.9, 2.5, 4.7, 4.9, and 3.3%, respectively, for zBW-EGG, zBW-PanUK, zBMI, zWC and zFM. After correcting through permutations, empirical *p*-values for the association of the best PRSs with phenotypic traits were 9.99E-05. The cross-correlation of the PRSs and phenotypic traits is shown in Figure 1. zBMI-related traits were correlated among them (r > 0.72) but not with zBW (r < 0.13). A similar pattern was observed for the PRSs but with a lower strength (r > 0.63, among PRSs for BMI-related traits; r = 0.48 among PRSs for zBW (EGG vs. PanUK); r <|0.06|, between PRSs for zBW and PRSs for BMI-related traits). The correlation coefficient between the traits and their PRSs ranged from 0.18 (zFM) to 0.29 (zBW).

### Association of the PRS and Maternal Smoking in Pregnancy With the Phenotypic Traits

The mean and SD of each trait, by the PRS group and by maternal smoking status during pregnancy, are shown in Supplementary Table S3. Children in the PRS-high group had higher phenotypic values than children in the PRS-mid group, which had higher values than children in the PRS-low group. Moreover, children exposed to maternal smoking presented lower zBW and higher values of the zBMI-related traits. To test the association of maternal smoking (any/sustained) and of the PRSs (continuous/three groups) with phenotypic traits, we fitted linear regression models adjusted for covariates.

In the model including the continuous PRS, any maternal smoking in pregnancy was at least marginally associated with lower child zBW and higher zBMI, zWC, and zFM (*p*-values <5.64E-02) **(** Table 2 **)**. The associations were only observed among children born from sustained smokers (*p*-values <2.99E-02) and not among children born of non-sustained smokers (*p*-values >0.402). In particular, children exposed to sustained smoking during pregnancy had 0.500 (standard error, SE = 0.100) lower zBW (adjusted for the PRS-PanUK), 0.321 (0.125) higher zBMI, 0.215 (0.099) higher zFM, and 0.280 (0.010) higher zWC than unexposed children. The effects of sustained maternal smoking on the phenotypic traits in the models including the PRS in three groups were similar **(** Table 2 **)**.

**TABLE 2:** Association of maternal smoking during pregnancy with phenotypic traits, adjusted for the PRSs and covariates.

	zBW (EGG)*			zBW (PanUK)**			zBMI			zFM			zWC		
	Effect	SE	*p*-value	Effect	SE	*p*-value	Effect	SE	*p*-value	Effect	SE	*p*-value	Effect	SE	*p*-value
Adjusted for the PRS (cont.)															
Any maternal smoking															
Non-smokers (ref.)	—	—	—	—	—	—	—	—	—	—	—	—	—	—	—
Smokers	−0.284	0.078	2.91E-04	−0.305	0.079	1.30E-04	0.206	0.099	3.84E-02	0.158	0.078	4.43E-02	0.151	0.079	5.64E-02
Sustained maternal smoking															
Non-smokers (ref.)	—	—	—	—	—	—	—	—	—	—	—	—	—	—	—
Non-sustained smokers	−0.049	0.123	6.92E-01	−0.06	0.124	6.28E-01	−0.009	0.155	9.56E-01	0.066	0.122	5.86E-01	−0.086	0.123	4.84E-01
Sustained smokers	−0.417	0.098	2.36E-05	−0.5	0.1	7.04E-06	0.321	0.125	1.06E-02	0.215	0.099	2.99E-02	0.28	0.01	5.08E-03
Adjusted for the PRS (3 groups)															
Any maternal smoking															
Non-smokers (ref.)	—	—	—	—	—	—	—	—	—	—	—	—	—	—	—
Smokers	−0.286	0.079	2.92E-04	−0.304	0.079	1.35E-04	0.208	0.1	3.70E-02	0.158	0.079	4.46E-02	0.158	0.079	4.65E-02
Sustained maternal smoking															
Non-smokers (ref.)	—	—	—	—	—	—	—	—	—	—	—	—	—	—	—
Non-sustained smokers	−0.045	0.123	7.15E-01	−0.061	0.124	6.24E-01	0.019	0.155	9.00E-01	0.067	0.122	5.85E-01	−0.067	0.123	5.84E-01
Sustained smokers	−0.428	0.099	1.64E-05	−0.445	0.1	8.37E-06	0.309	0.126	1.40E-02	0.213	0.099	3.20E-02	0.281	0.1	5.10E-03

Models were adjusted for the first 10 GWAS PCs and maternal education. Sample sizes are N = 1,080 for zBW, N = 1,063 for zBMI, N = 1,060 for zWC, and N = 1,052 for zFM.

*The PRS used in the models was calculated using the base GWAS from the EGG consortium.

**The PRS used in the models was calculated using the base GWAS from the PanUK Biobank.

In the model including sustained maternal smoking, the best PRSs (treated in continuous) were positively associated with the respective phenotypic traits (*p*-values <5.55E-08) (Table 3). The same pattern was observed when PRSs were categorized into three groups: low (<25% percentile), intermediate (25–75% percentile), and high (>75% percentile). Children in the PRS-high group of each trait had 0.634 (0.081) higher zBW (PRS-EGG), 0.538 (0.082) higher zBW (PRS-PanUK), 0.743 (0.103) higher zBMI, 0.451 (0.071) higher zFM, and 0.524 (0.080) higher zWC than children in the PRS-low group. Effect sizes remained similar when the models were adjusted for any maternal smoking during the pregnancy instead of sustained maternal smoking. Being above the 75th percentile of the PRS compared to being below the 25th percentile had a stronger effect (in absolute terms) than being exposed to maternal smoking during the whole pregnancy: 0.538 (PRS-PanUK) vs. −0.428 for zBW and 0.743 vs. 0.309 for BMI.

**TABLE 3:** Association of the PRSs with phenotypic traits, adjusted for maternal smoking during pregnancy and covariates.

	zBW (EGG)*			zBW (PanUK)**			zBMI			zFM			zWC		
	Effect	SE	*p*-value	Effect	SE	*p*-value	Effect	SE	*p*-value	Effect	SE	*p*-value	Effect	SE	*p*-value
Adjusted for any maternal smoking															
*PRS (cont.)*	0.241	0.028	5.82E-17	0.180	0.029	8.60E-10	0.259	0.037	3.85E-12	0.157	0.029	4.53E-08	0.190	0.028	3.39E-11
*PRS (3 groups)*
PRS-low (ref.)	—	—	—	—	—	—	—	—	—	—	—	—	—	—	—
PRS-mid	0.280	0.068	4.09E-05	0.235	0.069	7.47E-04	0.378	0.086	1.13E-05	0.134	0.067	4.82E-04	0.373	0.068	4.57E-08
PRS-high	0.627	0.080	1.26E-14	0.529	0.082	1.38E-10	0.707	0.103	1.29E-12	0.458	0.079	4.61E-09	0.526	0.08	6.54E-11
Adjusted for sustained maternal smoking															
*PRS (cont.)*	0.242	0.028	6.20E-17	0.182	0.03	8.01E-10	0.261	0.037	2.98E-12	0.157	0.029	5.55E-08	0.19	0.029	4.24E-11
*PRS (3 groups)*
PRS-low (ref.)	—	—	—	—	—	—	—	—	—	—	—	—	—	—	—
PRS-mid	0.275	0.068	5.76E-05	0.242	0.069	4.96E-04	0.382	0.086	1.02E-05	0.223	0.067	9.55E-04	0.362	0.07	1.17E-07
PRS-high	0.634	0.081	8.76E-15	0.538	0.082	7.60E-11	0.743	0.103	9.96E-13	0.451	0.071	1.75E-08	0.524	0.08	8.93E-11

Models were adjusted for the first 10 GWAS PCs and maternal education. Sample sizes are N = 1,080 for zBW, N = 1,063 for zBMI, N = 1,060 for zWC, and N = 1,052 for zFM.

*The PRS used in the models was calculated using the base GWAS from the EGG consortium.

**The PRS used in the models was calculated using the base GWAS from the PanUK Biobank.

### Gene by Environmental (GxE) Interaction

Then, we tested the interaction between maternal smoking during pregnancy and the best PRS for each trait in the association with the phenotype. We observed a GxE interaction for zBW between maternal smoking during pregnancy and the PRS-EGG treated in three groups (*p*-values interaction = 0.052 and 0.01, for any and sustained smoking, respectively) (Supplementary Table S4 and 5)**.** Maternal smoking during the whole pregnancy was associated with decreased zBW in children who had a certain genetic predisposition to low zBW (-0.399 (0.173) and -0.655 (0.157) for children in the PRS-low and in the PRS-mid groups, respectively). In contrast, the effect of sustained smoking was negligible in children within the PRS-high group (-0.097 (0.195)) (Table 4). However, this interaction was not observed when we analyzed the PRS-PanUK (*p*-value interaction = 0.096 and 0.341, for any and sustained smoking, respectively) (Table 4 **,** Supplementary Table S4 and 5). Finally, there was no evidence for a GxE interaction for zBMI-related traits (*p*-values for interaction >0.237) (Supplementary Table S4 and 5).

**TABLE 4:** Association of sustained maternal smoking status with zBW, by PRS group.

Trait	zBW (EGG)*			zBW (PanUK)**		
	Effect	SE	*p*-value	Effect	SE	*p*-value
PRS-low						
Non-smokers (ref.)	—	—	—	—	—	—
Non-sustained smokers	0.393	0.23	8.85E-02	0.067	0.231	7.70E-01
Sustained smokers	−0.399	0.173	2.19E-02	-0.215	0.188	2.55E-01
PRS-mid						
Non-smokers (ref.)	—	—	—	—	—	—
Non-sustained smokers	−0.389	0.187	3.79E-02	−0.104	0.19	5.85E-01
Sustained smokers	−0.655	0.157	3.70E-05	−0.584	0.146	7.53E-05
PRS-high						
Non-smokers (ref.)	—	—	—	—	—	—
Non-sustained smokers	0.02	0.235	9.32E-01	−0.098	0.243	6.87E-01
Sustained smokers	−0.097	0.195	6.21E-01	−0.421	0.213	4.93E-02

Models were adjusted for the first 10 GWAS PCS and maternal education. Sample sizes are N = 275 for PRS-low, N = 539 for PRS-mid, and N = 266 for PRS-high.

*The PRS used in the models was calculated using the base GWAS from the EGG consortium.

**The PRS used in the models was calculated using the base GWAS from the PanUK Biobank.

### Sensitivity Analyses

We, then, performed a series of sensitivity analyses. First, we repeated the analyses of sustained maternal smoking in relation to zBW without INMA and MoBa cohorts which are present in the EGG-based GWAS summary statistics (Supplementary Table S6), and the GxE interaction was still observed with a similar magnitude of the effect. The *R* ^2^ values explained by the PRS-EGG in the subsets of children without overlap were 4.8% (without INMA) and 4.2% (without INMA and MoBa). Second, we repeated the analyses of sustained maternal smoking in relation to zBW without preterm children, and the results did not change substantially (Supplementary Table S7). Third, the models of zBMI-related traits were analyzed again without adjustment for childhood second-hand smoke or restricting the analysis to children unexposed to second-hand smoke (Supplementary Table S8). Effect sizes did not change much in both cases; however, the associations of sustained maternal smoking during pregnancy were not significant any more in the restricted population, likely to the reduced sample size (n = 672).

## Discussion

In this study, we investigated the association of maternal smoking during pregnancy and genetic predisposition, independently and in combination, with BW and BMI-related traits measured in 1,086 European ancestry children of the HELIX project.

We found that mothers who smoked had newborns with lower BW and with higher BMI, WC, and FM in childhood, after adjusting for their corresponding PRS. The association between maternal smoking and BMI-related traits had been previously reported in HELIX publications of the early-life exposome, where maternal smoking was classified as non-smoking, passive smoking, and active smoking at some point during pregnancy (Vrijheid et al., 2020; Agier et al., 2021). In this study, we additionally explored the effect of the duration of smoking during pregnancy. We observed that the effects were stronger in children of sustained smokers, that is, mothers who smoked until the third trimester of pregnancy, compared to children of non-sustained smokers. Overall, our results are in line with the literature suggesting that maternal smoking reduces BW (Timmermans et al., 2014; Abraham et al., 2017; Kataoka et al., 2018; Chattrapiban et al., 2020) and increases BMI, total FM, and WC later in life (Behl et al., 2013; Timmermans et al., 2014; Magriplis et al., 2017; Rayfield and Plugge, 2017).

We calculated 10 PRSs for each one of the four traits of interest based on different *p*-value thresholds (Pt) of the base GWAS using PRSice v2, which is one of the most widely used tools to compute PRSs. The best PRSs were robustly associated with the phenotypic traits but only explained ∼4% of the phenotypic variation (*R* ^2^). In HELIX, the variation explained by the PRS of BMI that included 60,993 SNPs (*R* ^2^ = 4.7%) was in the range or slightly lower than previous estimations in children (R^2^ = 3%, 2 M SNPs (Odintsova et al., 2021); *R* ^2^ = 11%, 2.1 M SNPs (Hüls et al., 2021)), in adolescents (*R* ^2^ = 6.5%, 941 SNPs; Xie et al., 2020), or in adult individuals (*R* ^2^ = 2.9%, 97 SNPs (Dashti et al., 2022); *R* ^2^ = 5.2%, 376 SNPs (Sulc et al., 2020); R^2^ = 6.7%, 2 M SNPs (Odintsova et al., 2021); *R* ^2^ = 7.8%, 2.1 M SNPs (Khera et al., 2019)). In Khera et al., children in the 10th percentile of the PRS for BMI, which included 2.1 M SNPs, weighed 3.5 kg more than children in the lowest percentile. The predictive power of that PRS was restricted to postnatal BMI from childhood to adulthood, with low predictability for BW. Similarly, in our data, the PRS of BMI was not correlated with the PRS of BW or vice-versa. In contrast, the PRSs of BMI-related traits (BMI, FM, and WC) were correlated among them, suggesting pleiotropic genetic effects underlying these phenotypic associations, as suggested earlier (Vogelezang et al., 2020; Dashti et al., 2022). In our study and using the PRSice v2 tool, the addition of more SNPs in the PRS of BMI did not increase the predictive power. As evidenced in the literature, the number of SNPs of the PRS not necessarily correlates with the predictability of the PRS, suggesting that other factors are also important. The slightly lower prediction of the PRS of BMI in our study compared to others might be due to the use of different base GWAS and/or different statistical methods to calculate the PRS. Moreover, it also could be explained by the less similarity between the genetic background of the base and target populations (HELIX is composed of European ancestry children from six different countries and the base GWASs were conducted in adults from the UK Biobank). To control for this, our models were adjusted for the first 10 GWAS PCs which also might have attenuated the associations.

Regarding BW, previous studies have reported that a PRS for BW including 62 SNPs from the EGG consortium explains between 2 and 4.9% of the phenotypic variation (Horikoshi et al., 2016). In HELIX, the PRS with 3,316 SNPs derived from EGG explained 4.9% of the BW variance. Odintsova et al. also computed a PRS for BW using the PanUK Biobank data (Odintsova et al., 2021). Their PRS that included 9 M SNPs explained 1.4% of the variance of BW, similar to the PRS-PanUK with 18,563 SNPs that explain 2.5% of the variance in HELIX. As for BMI, the PRS for BW do not show a linear relationship between the number of SNPs and the variance explained.

For all the traits, having a PRS above the 75th percentile (PRS-high) compared to having a PRS below the 25th percentile (PRS-low) had a stronger effect (in absolute terms) than the exposure to tobacco smoke during the whole pregnancy. Children with a PRS-high level weighed ∼266 g more at birth and had ∼1.573 kg/m^2^ more in childhood than children in the PRS-low. Offspring born from sustained smoker mothers had a mean reduction of BW of ∼173 g and a mean increase of BMI of ∼0.742 kg/m^2^, compared to offspring of non-smokers. Of note, the comparison of effect sizes between the PRS and maternal smoking is subjected to the PRS categorizations we applied (i.e., above 75th percentile vs. below 25th percentile) and to measurement error, which is likely larger for maternal smoking (i.e., self-reported information).

The GxE analyses showed low evidence of interactive effects for BMI-related traits between the PRSs and maternal smoking during pregnancy. In contrast, other studies have reported GxE interactions between BMI genetic predisposition with a PRS including 2.1 M SNPs, and geographic region in Europe, parental education, fiber intake, or screen time (Hüls et al., 2021), and between a PRS with 97 SNPs and obesity lifestyle risk index that includes alcohol intake, education, exercise, sleep habits, shift work, and own smoking (Dashti et al., 2022). In addition, a recent study, based on a new statistical method that does not require environmental factors to be measured, has reported that GxE interactions account for 1.9% of the BMI variance, while the PRS alone for 5.2% (Sulc et al., 2020).

We detected an interaction for BW between sustained maternal smoking and the PRS-EGG, which was not confirmed when using the PRS-PanUK. Compared to others, children with a genetic predisposition to high BW (EGG PRS-high group) seemed to be protected against the adverse effects of sustained maternal smoking on BW. Surprisingly, the adverse effect of maternal smoking on BW was stronger in children in the PRS-mid group than in children in the PRS-low group, likely due to the low numbers in each group. The interaction did not reach statistical significance when treating the PRS in continuous. Moreover, despite the correlation between the two PRS for BW (r = 0.48), the protective effect of the genetic background was not observed in children in the PRS-high group computed with the PanUK base GWAS. The EGG base GWAS data includes around 25% of the HELIX children, which represent <0.2% of the base sample (Warrington et al., 2018). This is known to introduce some inflation in the predictability of the PRS, which is proportional to the fraction of the target sample that overlaps the base sample (Choi et al., 2020). We estimated that the overlap of samples between the base and target population would produce a false positive rate of ∼35% (Choi et al., 2021). In order to address this limitation, we repeated the analysis without children potentially included in the EGG base GWAS, and the results did not change, suggesting the inflation due to the overlap of individuals has a minor impact on the findings. On the other hand, the lack of effect modification of the PRS-PanUK could be explained by the lower variability it captures compared to the PRS-EGG (2.5% compared to 4.9%). As far as we know, there are no other studies exploring the interaction of these PRSs with maternal smoking during pregnancy for comparison. Thus, validation of these findings in other studies is necessary.

The results should be considered in light of some limitations. The first one is that PRS predictability depends on the quality of the reference GWAS (sample size, phenotype definition, ancestry, and base-target sample overlap). Second, due to the lack of reference GWAS in non-European populations, we only calculated the PRSs in European ancestry HELIX children. Third, HELIX is a pediatric population composed of children from 5 to 12 years, while the base GWAS for BMI-related traits were conducted in adults. Hence, genetic variants having specific effects only in adults might have biased our findings, while variants having specific effects only in children, and thus not considered in the PRS, might have decreased the PRS predictability. In any case, our PRSs could predict part of the variation of the traits, indicating that some genetic factors seem to be stable over life, as described before for BMI (Vogelezang et al., 2020). Fourth, maternal genotypes were not available to study their contribution to the phenotypes, especially on BW (Warrington et al., 2019). Fifth, as PRSs explain a small percentage of the variation of the trait, they might not be sufficient to test GxE interactions in our relatively small sample size, and large sample sizes will be required. However, this approach is still more powerful than testing GxE interactions with single SNPs. Finally, genetic variants in genes involved in different biological pathways were combined to compute the PRSs. This might have limited the identification of interactions within a specific biological pathway affected by the exposure (i.e., inflammation and glucose metabolism). In the future, it would be interesting to create pathway-specific PRSs of each trait and test their interaction with maternal smoking.

Nonetheless, our study also has some strengths. First, HELIX is a well-characterized cohort in which phenotypic measurements were obtained under extremely harmonized protocols. Second, we have focused on maternal smoking during pregnancy, a well-characterized exposure for which strong effects on offspring health outcomes have been described. Finally, this study could serve as a basis for larger studies combining data of different cohorts that aim to investigate GxE interactions.

In summary, maternal smoking during the whole pregnancy period, but not only at the beginning, was related to lower BW and higher BMI-related traits in childhood. The PRSs were associated with the phenotypic traits, but they explained a low proportion of the variation. There was low evidence for GxE interactions, except for BW, where children in the highest PRS group seemed to be protected against the damaging effects of sustained maternal smoking. However, this was not validated when using another PRS for BW, and thus, it requires further investigation.

## Data Availability

The raw data supporting the current study are available from the corresponding author on request subject to ethical and legislative review. The “HELIX Data External Data Request Procedures” are available with the data inventory in this website: http://www.projecthelix.eu/data-inventory.
