# Supplementary material for: Study of the Combined Effect of Maternal Tobacco Smoking and Polygenic Risk Scores on Birth Weight and Body Mass Index in Childhood
Source: Front Genet. 2022 May 12;13:867611. doi: 10.3389/fgene.2022.867611 (PMC9133473; doi:10.3389/fgene.2022.867611)
Supplement: Supplementary file 2 [file DataSheet1.docx]

**Combined effect of maternal tobacco smoking and polygenic risk scores on birth weight and body mass index in childhood**

**Supplementary Figures**

HELIX children with genetic data

**N=1,304**

Number of individuals for validation within PRSice:

zBW

**N=1,119**

zBMI

**N=1,155**

zWC

**N=1,152**

zFM

**N=1,143**

Exclusion of children of non-European ancestry

**N=149**

HELIX children of European ancestry with genetic data

**N=1,155**

Exclusion of children without information of at least one phenotype, maternal smoking and main covariates

**N=69**

HELIX children of European ancestry with maternal smoking and maternal education (main database):

**N=1,086**

Samples size after adjusting for covariates, by trait:

Pregnancy: maternal smoking, maternal education and 10 GWAS PCs

zBW

**N=1,080**

Postnatal: maternal smoking, maternal education, second-hand smoke and 10 GWAS PCs

zBMI

**N=1,063**

zWC

**N=1,060**

zFM

**N=1,052**

Adjusted for GWAS PCs, for gestational age and sex (zBW) or for second-hand smoke (zBMI, zWC and zFM).

**Figure S1.** Flow chart illustrating the selection of children for the study. Solid lines and boxes represent individuals remaining for the study while dashed lines and triangles depict excluded individuals. Exclusion reasons as well as the total of individuals are indicated in each box and triangle. Initial sample size includes 1,304 children with genetic data among the >32,000 children in the HELIX project.


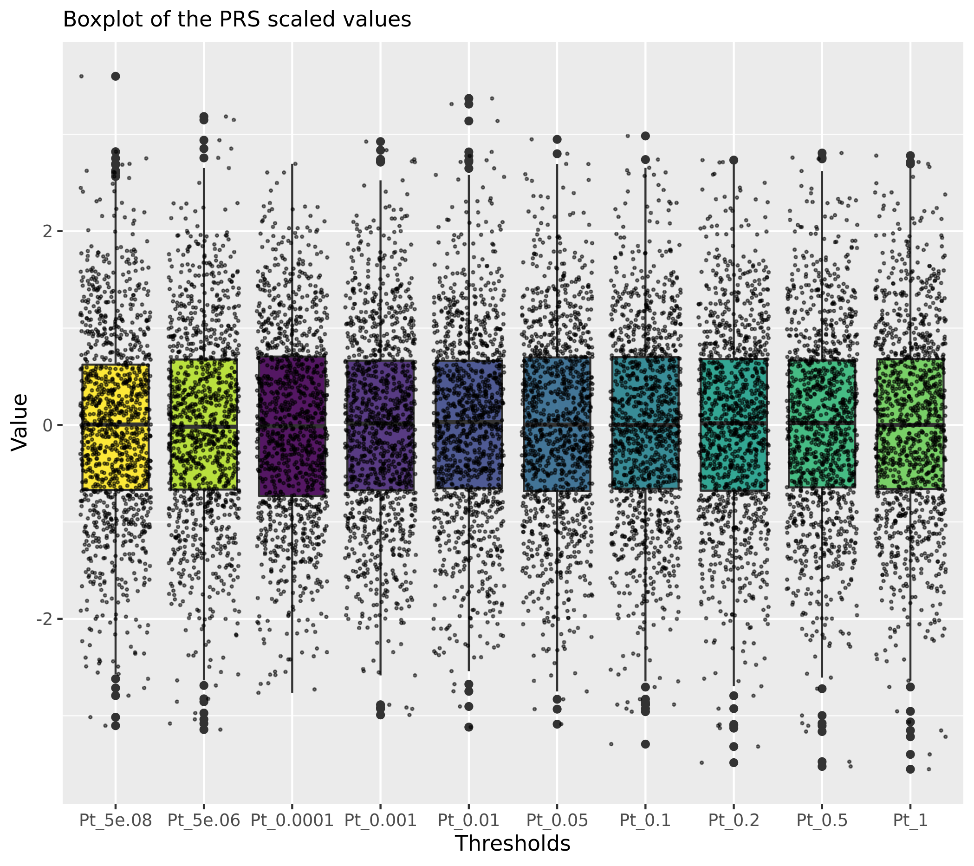
A1)

A2) A3)


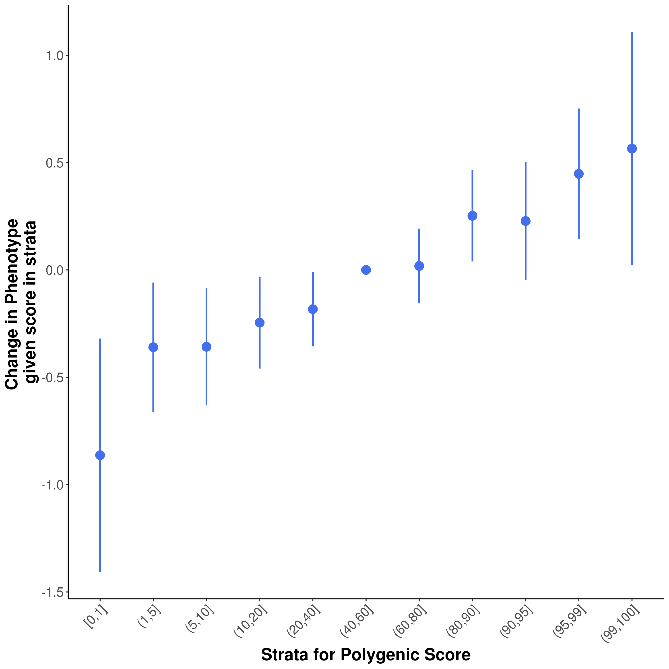

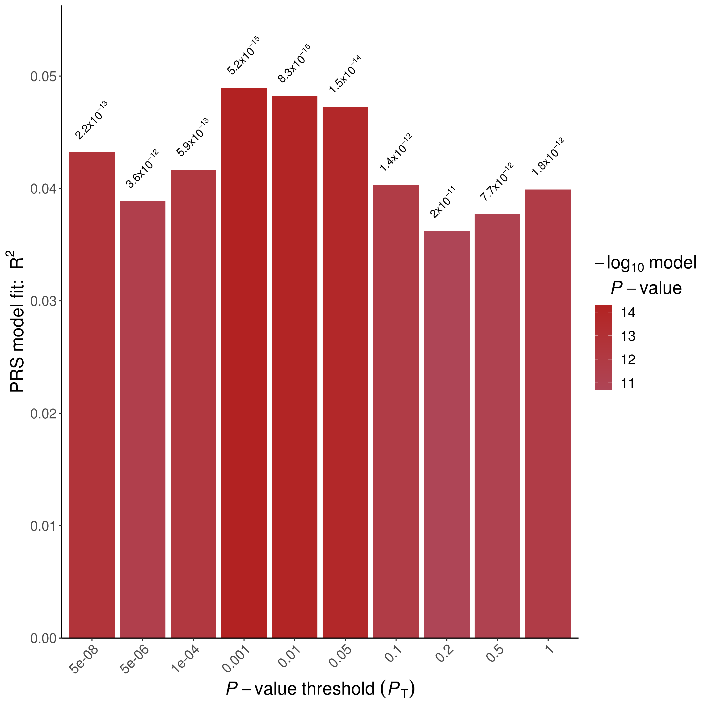


B1)


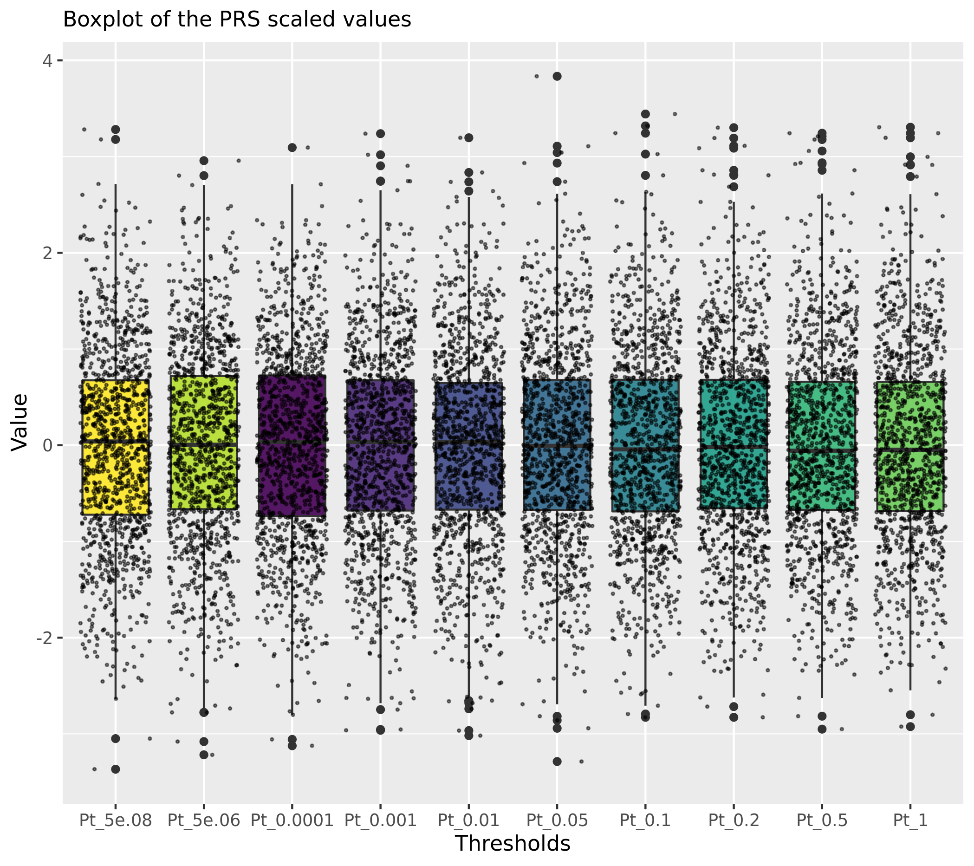


B2) B3)


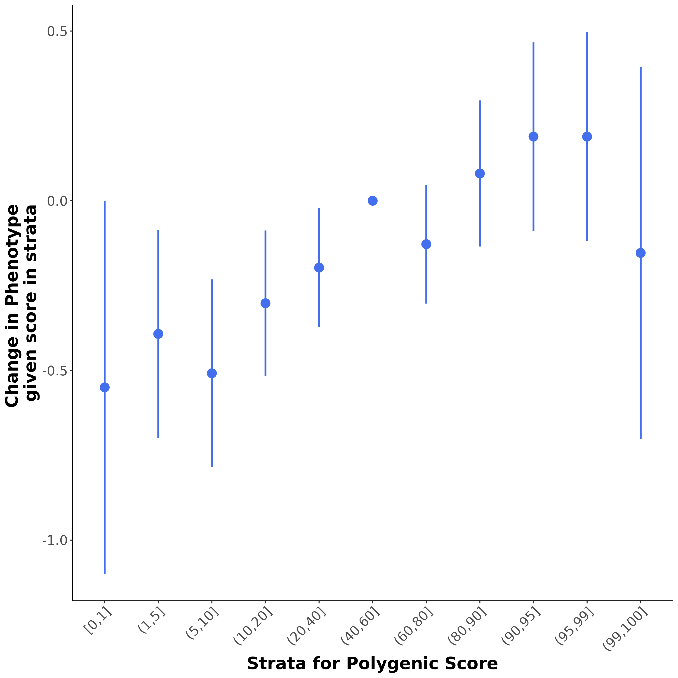

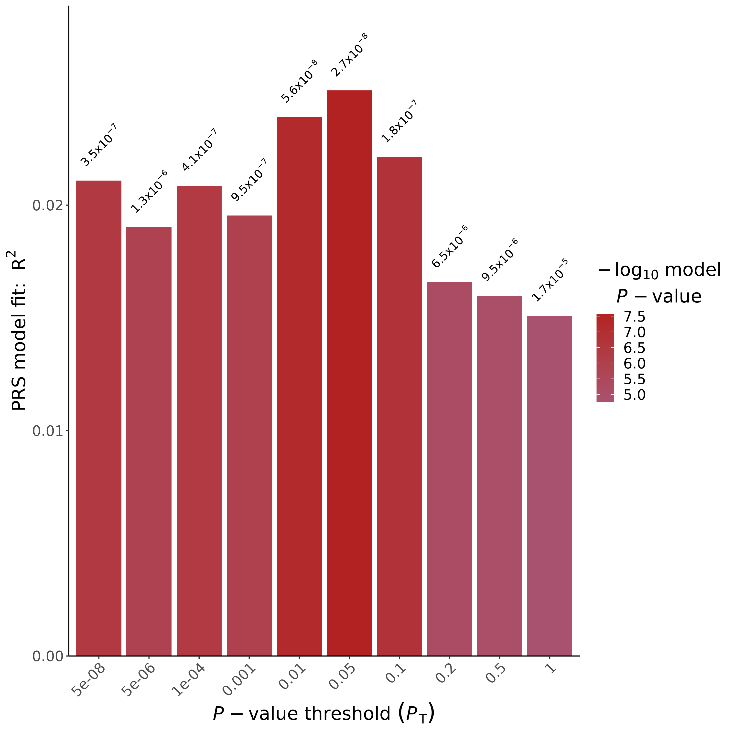


C1)


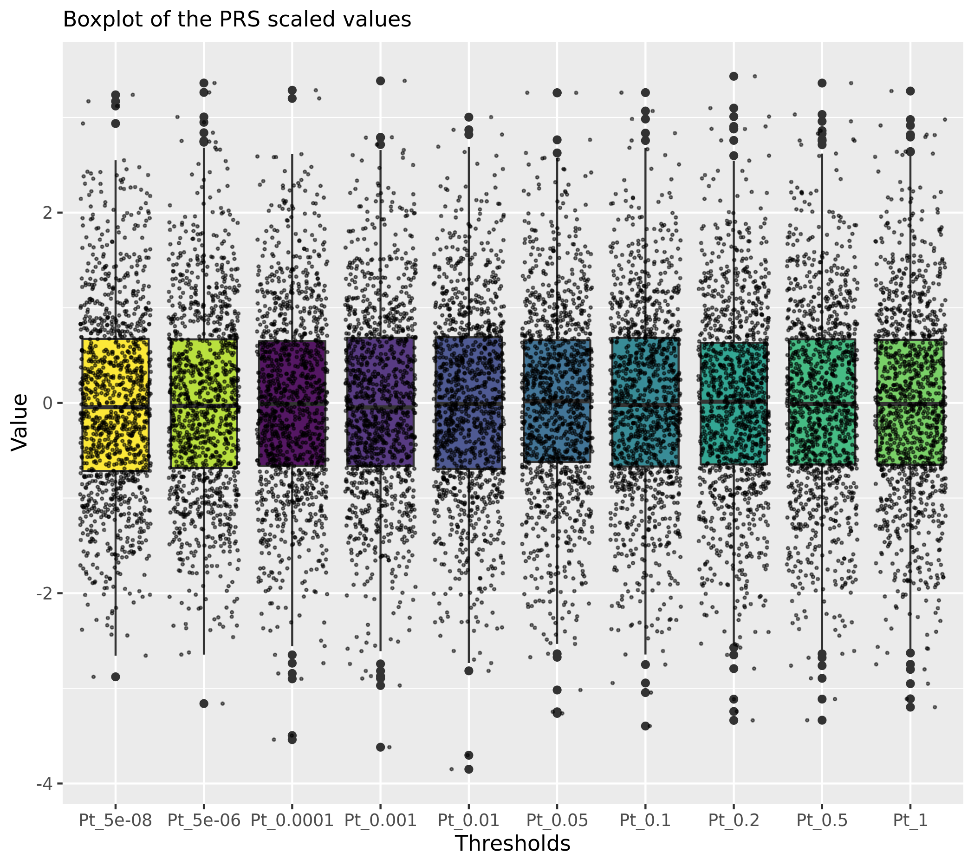


C2) C3)


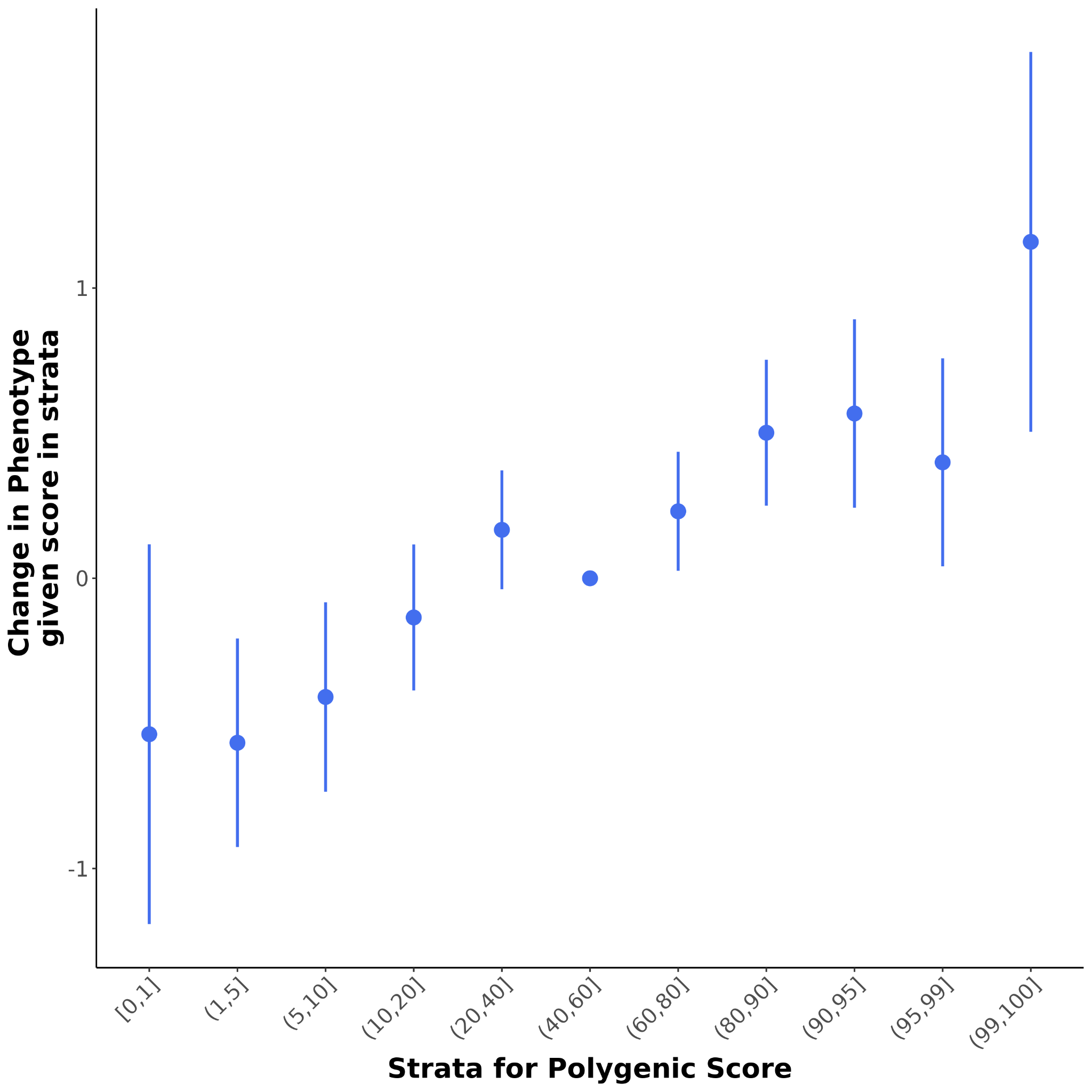

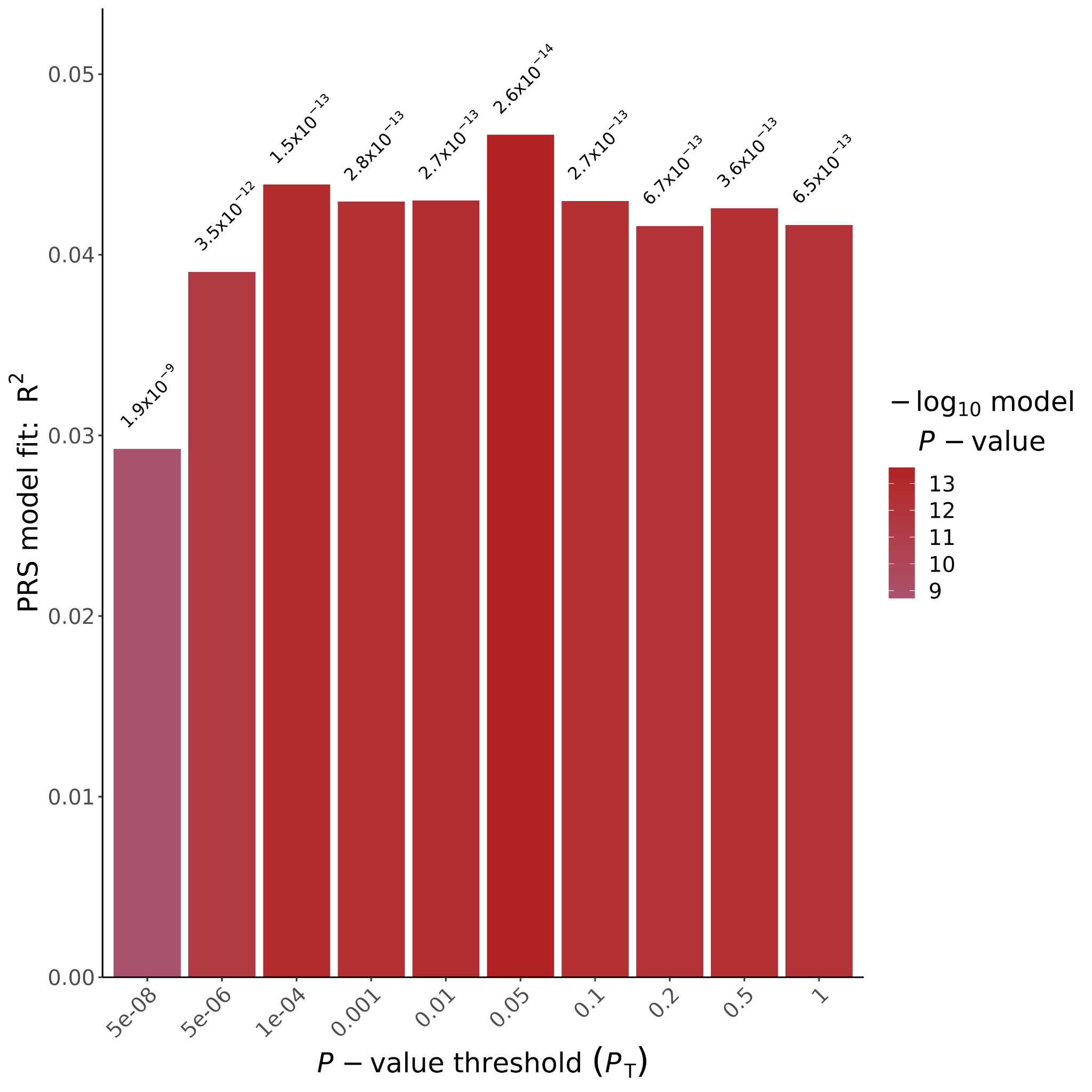


B3)

D1)


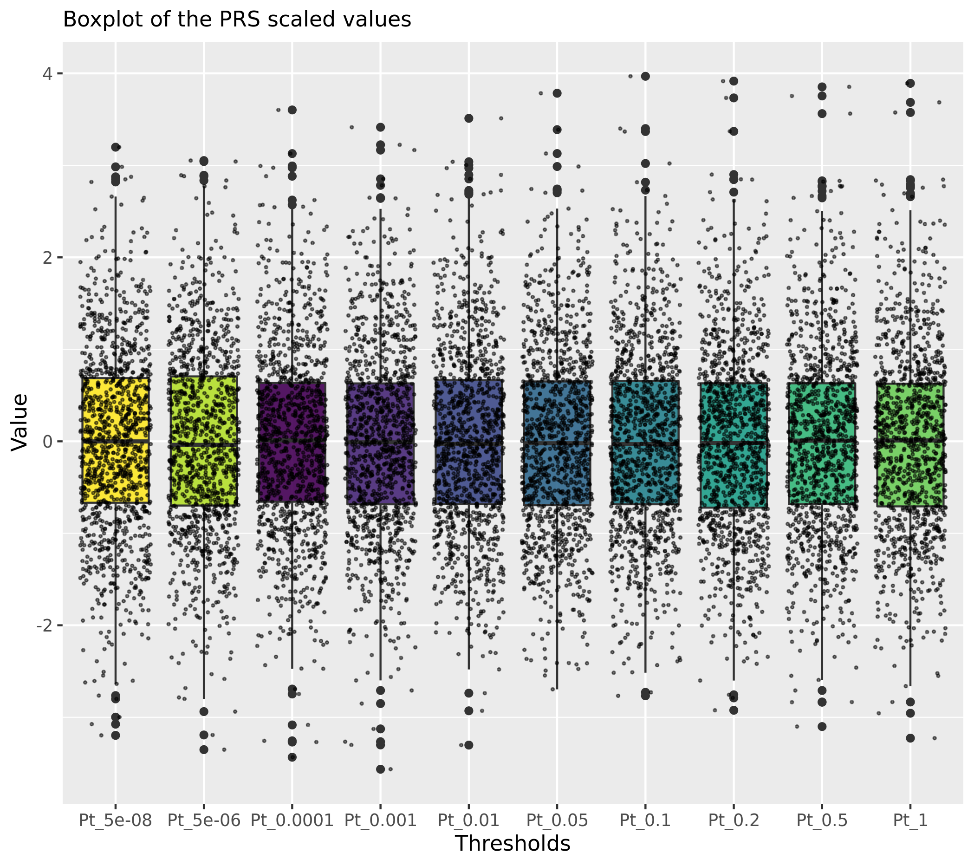


D2) D3)
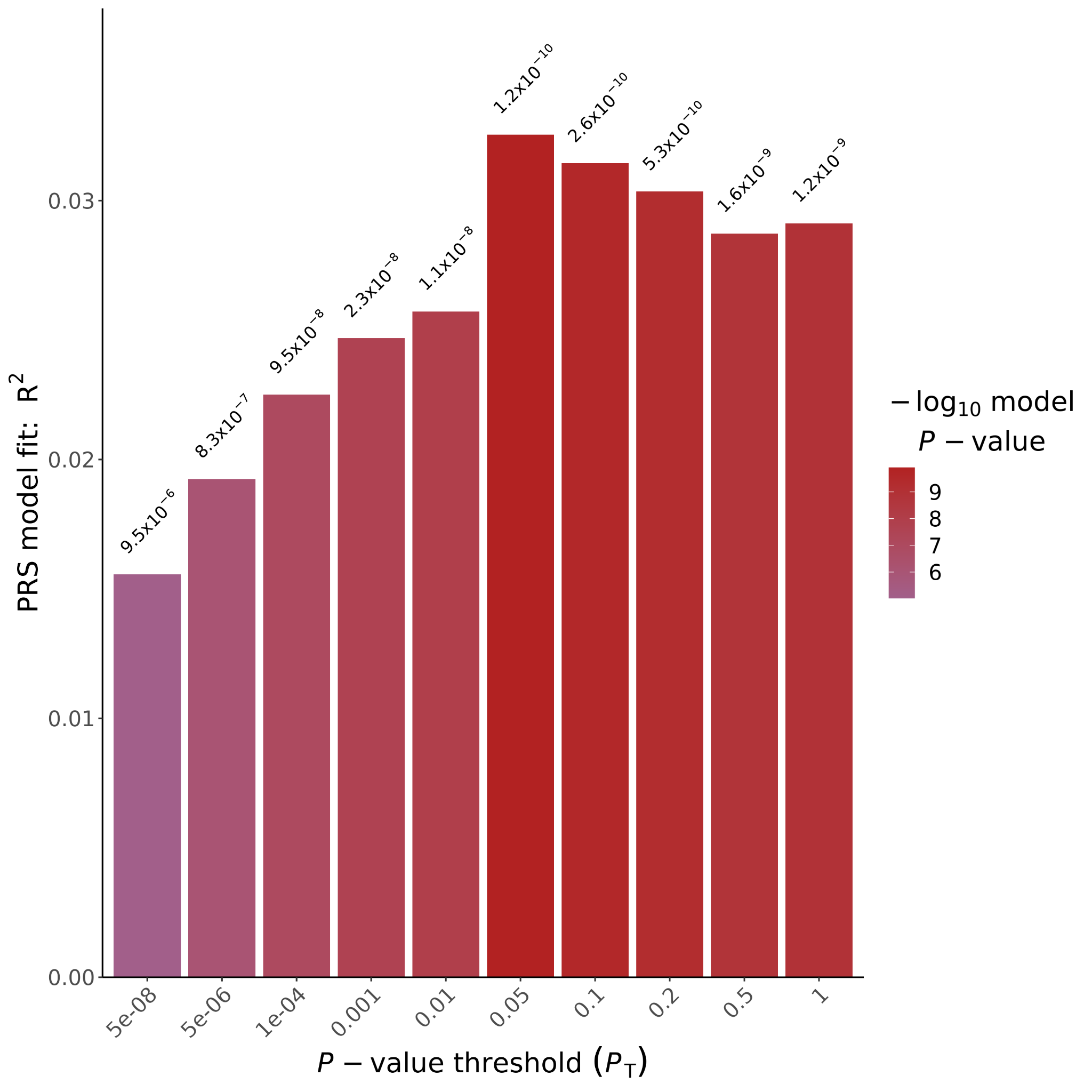


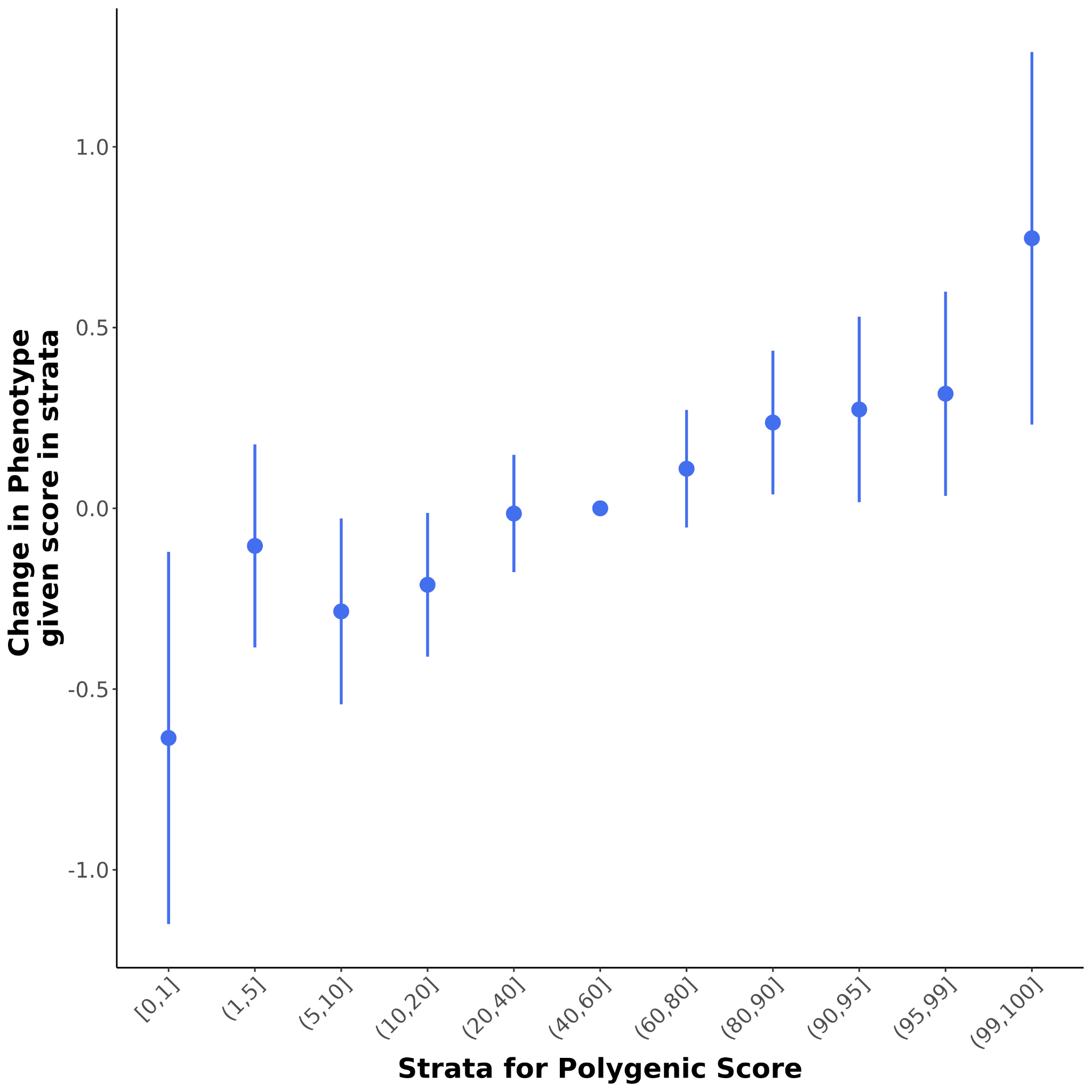


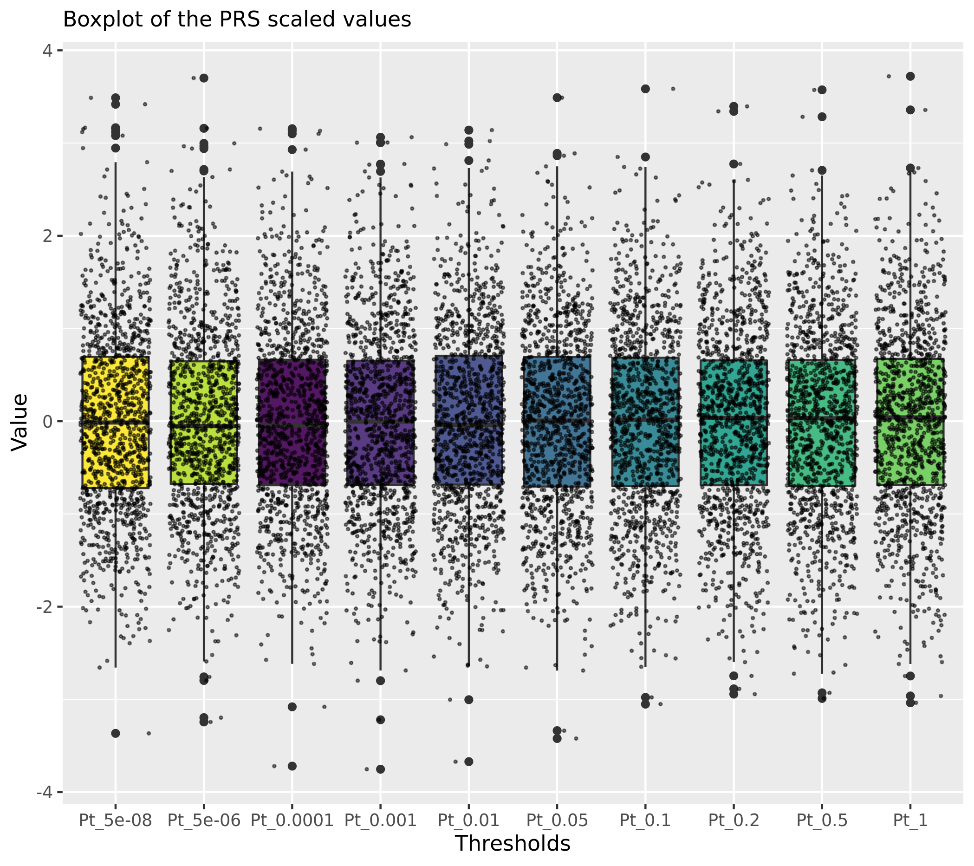
E1)

E2) E3)
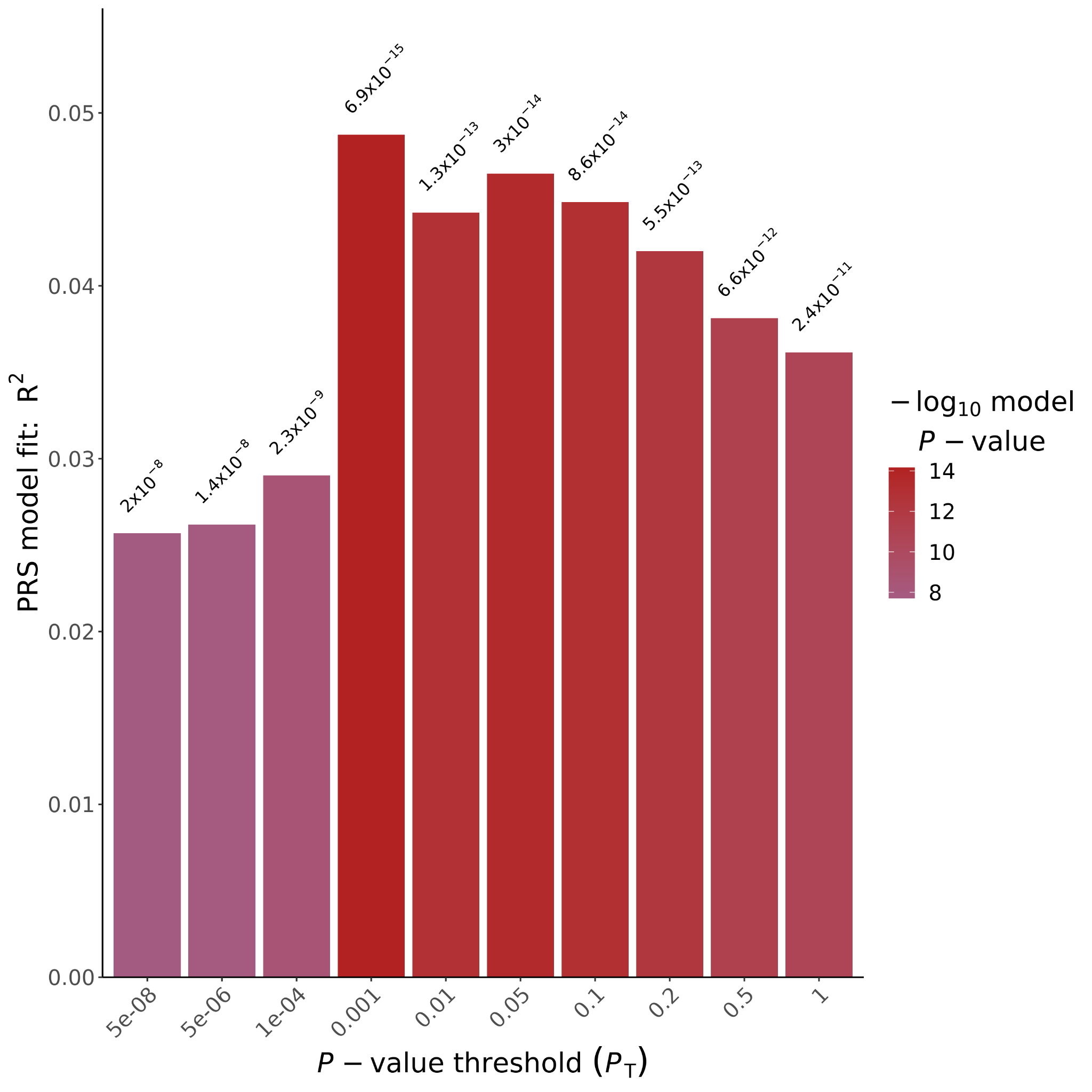

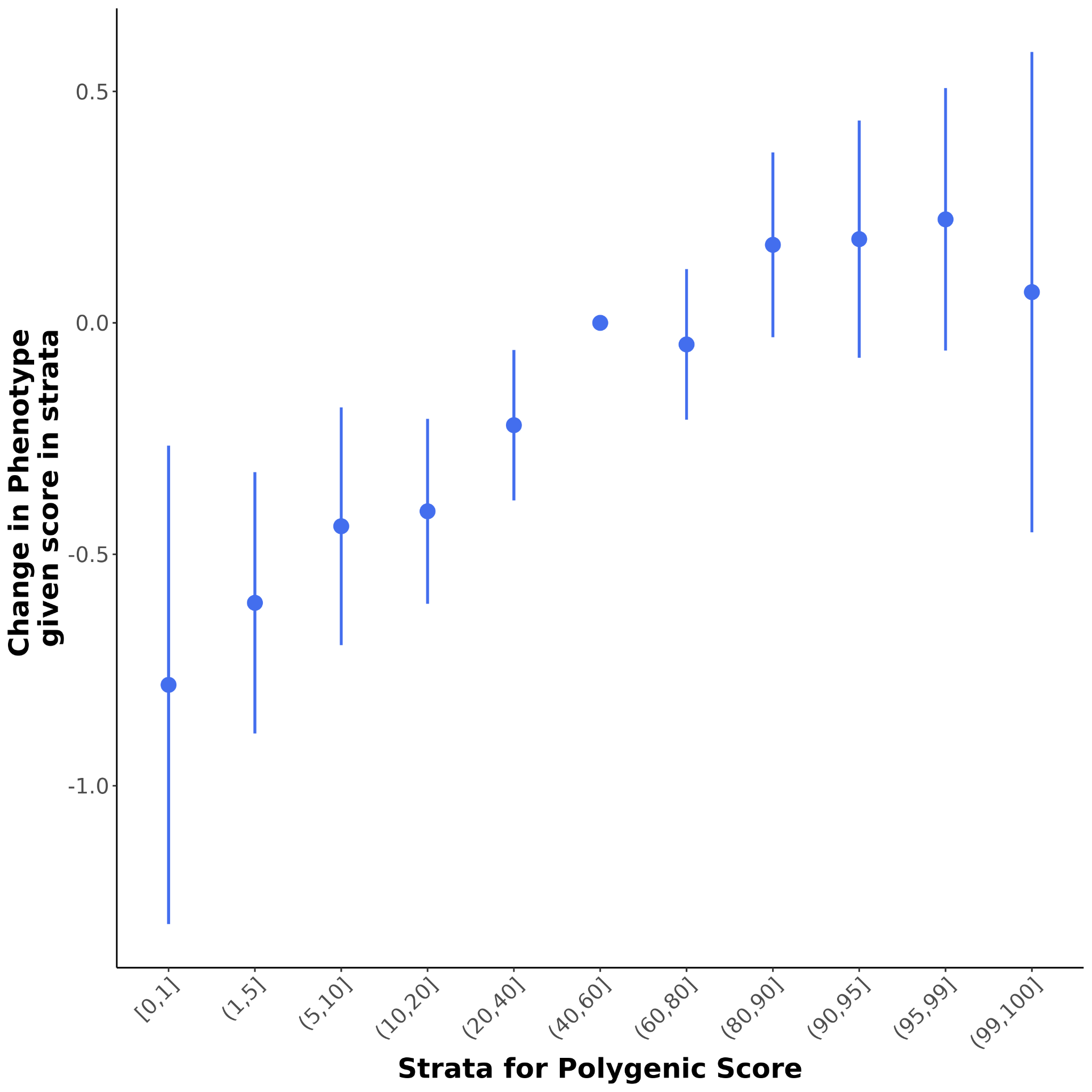


**Figure S2**. Summary plots of the PRSs for zBW using base GWAS form Early Growth Genetics (EGG) consortium (A), zBW using base GWAS form PanUK Biobank (B), zBMI (C), zWC (D), and zFM (E). 1) Distribution of the 10 PRSs (scaled) for the 1,155 HELIX children. 2) Variance of the phenotypic trait (R^2^) explained by the 10 PRSs (not scaled), after adjusting for the 10 first GWAS PCs. The x-axis represents the 10 PRSs and the y-axis the R^2^. 3) Association of the best PRS (not scaled) with the phenotypic trait, after adjusting for the 10 first GWAS PCs. The x-axis represents the PRS divided in 10 quartiles and the y-axis the phenotypic trait. Best PRS is defined as the PRS with the highest R^2^. Sample sizes of the association analysis are shown in Figure S1.
